# The Association Between Intimate Partner Encouragement of Alcohol Use and Alcohol Use Among Females Formerly Involved in the Juvenile Justice System

**DOI:** 10.1007/s10935-025-00828-z

**Published:** 2025-02-15

**Authors:** Avery Turner, Diana Jenkins, Maria Schweer-Collins, Leslie D. Leve

**Affiliations:** 1 College of Education, University of Oregon, Eugene, USA; 2 Prevention Science Institute, University of Oregon, Eugene, USA; 3 Department of Psychology, Iowa State University, Ames, USA

**Keywords:** Romantic partner, Intimate partner, Alcohol use, Emerging adulthood, Juvenile justice, Female

## Abstract

Females who are involved with the juvenile justice system as adolescents are at risk for heavy alcohol use, which is associated with an increased risk of recidivism and negative health outcomes. Alcohol use peaks during emerging adulthood and intimate partners play an increasingly important role in decision making during this developmental period. Using data from a longitudinal study of females who were involved in the juvenile justice system as adolescents (*n* = 80), we investigated whether intimate partner encouragement of alcohol use is associated with higher rates of alcohol use frequency among this population as they enter emerging adulthood. Participants reported on their partners’ encouragement of their alcohol use at an in-person study visit when they were emerging adults, and then reported on their own alcohol use approximately six months later. A negative binomial regression was fit to the data and estimated that for each standard deviation increase in reported intimate partner encouragement of alcohol use, participants’ rate of alcohol consumption increased by 109% six months later. These findings indicate that intimate partner encouragement of alcohol use may be a risk factor for heavy drinking among emerging adult females with a history of chronic and severe delinquency. These findings have implications for prevention, as they indicate a need to measure intimate partner encouragement of alcohol use in studies that involve high-risk emerging adult females and may suggest that intimate partners should be included in interventions intended to reduce heavy alcohol use.

Excessive alcohol use in women is associated with an increased risk of liver disease, cognitive decline, damage to the heart muscles, and cancer, and these negative health effects develop quicker and at higher rates than for men (Center for Disease Control and Prevention, 2022b). Females involved with the United States (U.S.) juvenile justice (JJ) system report high rates of alcohol use (Staton et al., 2020) and are more likely than their male counterparts to report being under the influence of alcohol and/or other drugs at the time of their last offense (48% vs. 43%; Sedlak & Bruce, 2016). While youth arrests have declined overall, the proportion of females in the U.S. JJ system has increased by nearly 50% in the past thirty years (Hockenberry & Puzzanchera, 2023). In a foundational study of psychiatric disorder prevalence rates among juvenile detainees in Cook County, Illinois, U.S., 20% of female detainees aged 10 to 18 met the criteria for an alcohol use disorder (AUD; Teplin et al., 2015). At follow-up interviews 3 and 4.5 years later, rates of AUD declined among these women, but remained nearly twice as high as the national average for women during the study follow-up period (1998–2003; Harford et al., 2005). Because females involved with the JJ system are at risk for developing AUD (Teplin et al., 2015), it is important to identify risk factors for heavy alcohol use for this population during emerging adulthood, the period between ages 18–29 (Arnett, 2014), since this developmental period is associated with the highest rate of heavy episodic drinking (Center for Disease Control and Prevention, 2022a).

Although prior research has focused on other risk factors that may be posed by intimate partnerships among females with JJ system involvement (Garcia & Lane, 2012), we postulate that intimate partner encouragement of alcohol use may pose an additional risk to this population for future increases in alcohol use. Using the Social Development Model, which theorizes that social bonds can influence individual behavior both positively and negatively (Catalano & Hawkins, 1996), we propose that intimate partner encouragement of alcohol use serves as a risk factor for future increases in alcohol use among females with prior JJ involvement.

There is a growing body of research identifying risk and protective factors for females with JJ system involvement (Leve et al., 2015), yet research on the role of intimate partner encouragement of alcohol use is scant. We identified only one study that had investigated the link between intimate partner encouragement of alcohol use and alcohol use among high-risk emerging adult drinkers (Cheong et al., 2021). Below we summarize longitudinal data that suggests that females with a history of criminal justice system involvement experience greater social costs due to their higher rates of substance use compared to their male counterparts (Estrada & Nilsson, 2012), explore theories that explain the link between partner- and self-alcohol use and potential moderators of that relationship (Fleming et al., 2010), and review prior research on the role of intimate partner encouragement of alcohol use among high-risk emerging adult drinkers (Cheong et al., 2021).

## Rates and Costs of Substance Use Among Women with Criminal Justice System Involvement

Longitudinal data from Sweden suggest that females with criminal justice system involvement use substances at higher rates than their male counterparts, and this may account for the higher social costs faced by females with criminal justice system involvement (Estrada & Nilsson, 2012). Data from over 14,000 participants who were born in Sweden in 1953 and followed until they were 48 years old revealed that a greater proportion of females with criminal justice system involvement engaged in substance misuse than males, but this may be due to the higher number of risk factors present among females in this population. The authors also described the higher social costs associated with criminal involvement for females; females with criminal involvement were more likely to live alone on little to no income as compared to non-criminal justice system involved females, and this discrepancy was larger for females than it was for males. Nearly two-thirds of females with repeated criminal involvement, termed “persisters”, were receiving welfare benefits or unemployed at age 48, compared to less than 40% of male persisters at the same age. This difference between female and male persisters was no longer significant when controlling for substance use, suggesting that the differential social costs of criminality may be due, in part, to differential levels of substance use. Although the nature of criminal behavior and substance use trajectories have likely changed since these data were collected, these longitudinal findings provide valuable insights into the differential effects of criminality among females compared to males.

In addition to the higher social costs that criminal justice system involved females face (Estrada & Nilsson, 2012), some research suggests that there is a stronger link between substance use and recidivism in females than there is for males (Andrews et al., 2012). Because females involved in the JJ system who use substances are more likely to re-enter the criminal justice system, there is an urgent need to address substance use among this population due to their higher rates of substance use and the associated costs.

## Romantic Relationships and Alcohol Use

Fleming et al. (2010) followed young adults from high school to two years post-high school to examine the relationship between substance use and romantic relationships. They found that young adults involved in romantic relationships were less likely to engage in heavy drinking, even when controlling for adolescent alcohol use, and that partner alcohol use was significantly associated with the participant’s alcohol use. A positive correlation between partner- and self-alcohol use was found in a prior analysis of the data that the present study will analyze (Rhoades et al., 2014). Fleming et al. (2010) summarized two theories to explain the correlation between partner- and self-alcohol use: young adults may be attracted to others who use alcohol in a way that is similar to them (“assortative mating”) or there may be a “contagion effect” wherein one partner’s alcohol use causes the other person to increase or decrease their use to match that of their partner (Fleming et al., 2010). The study also found an interaction effect of partner alcohol use on relationship quality and the degree to which the young adult’s drinking behavior matched that of their partner; as relationship quality improved, the likelihood of heavy drinking decreased for young adults whose partners didn’t/rarely drank and increased for those whose partners drank heavily (Fleming et al., 2010). While Fleming et al. (2010) investigated the effect of partner substance use on participant substance use more broadly, we posit that a unique mechanism of influence is active encouragement of alcohol use by an intimate partner.

### Intimate Partner Encouragement of Alcohol Use

There is limited research exploring the role of intimate partner encouragement of alcohol use on emerging adult alcohol use. In a community-based sample of emerging adults who met the criteria for high-risk drinking, Cheong et al. (2021) found that peer discouragement of alcohol use was associated with reduced alcohol consumption, but there was no effect of intimate partner discouragement. This study measured discouragement by asking respondents a single item about how much their partner discouraged their drinking from “not at all” to “a great deal,” which may not adequately capture all dimensions of discouragement. Because the Cheong et al. (2021) study captured the participant’s report of discouragement from their partners at the same time as they measured alcohol use, it is unclear whether the intimate partner discouragement preceded the alcohol use or if this sample’s high-risk drinking was the cause of their partner’s discouragement. Also, this study did not specifically recruit individuals from vulnerable populations, and their findings may not generalize to emerging adult females formerly involved in the JJ system. Furthermore, partner encouragement of alcohol use may not be the direct opposite of partner discouragement.

Although the Cheong et al. study (2021) has limitations, the authors emphasized the importance of utilizing a community-based sampling method given that the vast majority of research on alcohol use has been done with college students. Because youth involved with the JJ system are less likely to enroll at a four-year college (Widdowson et al., 2016), females involved with the JJ system are likely underrepresented in alcohol use research. Therefore, it is important to identify risk and protective factors for this population given that existing evidence-based interventions for alcohol use may not be validated for them.

## **Social Development Model as a Guiding Theory**

The Social Development Model (SDM) synthesizes control theory, social learning theory, and differential association theory to explain behaviors such as drug use from a developmental perspective (Catalano & Hawkins, 1996). SDM theorizes that social bonds can serve as protective or risk factors for individuals, depending on the behavioral norms of the person to whom the individual is bonded. SDM recognizes that social influences change in salience throughout different developmental periods. This model is useful when investigating risk and protective factors for females because previous research has found that females may be influenced by their partners’ health-harming behaviors to a greater extent than males (Moretti et al., 2004). Although several theories have been put forth to explain the correlation between partner- and self-alcohol use (Fleming et al., 2010), the role of intimate partner encouragement of alcohol use has not been fully investigated. Using the SDM to guide our theory that intimate partners are capable of influencing their partner’s alcohol use, we identified intimate partner encouragement of alcohol use as a specific mechanism of risk for future increases in alcohol use among this population.

This study adds to the existing literature by investigating the role of intimate partner encouragement of alcohol use in a high-risk sample by capturing data on various aspects of encouragement of alcohol use. Furthermore, we examine the role of intimate partner encouragement of alcohol use among a sample of females formerly involved with the JJ system who are underserved and for whom the role of intimate partner encouragement of alcohol use on alcohol use outcomes has not been explored. Given that females are influenced by their partners’ health behaviors (Moretti et al., 2004; Catalano & Hawkins, 1996), we hypothesized that intimate partner encouragement of alcohol use would be associated with greater alcohol use frequency during emerging adulthood.

## Methods

### Participants and Procedures

The current study consists of secondary data analysis from a study of *N* = 166 female adolescents with severe and chronic delinquency who participated in a longitudinal study that investigated the efficacy of Treatment Foster Care Oregon (TFCO; see Leve et al., 2022 for more details). Participants were randomly assigned to TFCO or usual group care services during adolescence. In TFCO, youth were placed individually with foster parents who were trained to provide the participant with close supervision, mentoring, and consistent limits. Participants were followed through emerging adulthood, when they provided data on their alcohol use and the encouragement of their alcohol use from their intimate partners. Recruitment for the original study took place in Oregon, US, between 1997 and 2006, and the emerging adulthood assessments occurred between 2009 and 2012. Participants were included in the current study if they indicated at the emerging adult interview (T2) that they: (1) used alcohol within the past six months, and (2) had an intimate partner.

Of the 166 original participants, 151 participated in the T2 interview (*n* = 10 of the original participants were not recruited, *n* = 3 did not complete the emerging adult interview, and *n* = 2 were deceased). Twenty-one of the 151 participants were excluded from the current study because they did not have a partner at the time of the emerging adult interview. Additionally, 50 of the 151 participants were excluded from the current study because they did not endorse drinking alcohol in the six months prior to the T2 interview, resulting in a final sample of 80 participants. Of those 50 excluded participants, at baseline, 13 reported that they had never drank, 6 had drank once or twice, 10 drank “occasionally”, 13 drank 1–6 times per week, 7 drank once or more per day, and 1 had a missing value for their baseline alcohol use. A Welch two sample t-test was run to ensure that participants who were included in this analysis (*n* = 80) did not have significantly different levels of baseline alcohol use than the participants who were excluded for reasons other than not drinking (*n* = 36). This test revealed that the included participants’ baseline alcohol use was not significantly different from the participants who were excluded for reasons other than not drinking at the time of the emerging adult interview (*M1* = 3.09, *M2* = 3.23, *t* = -0.52, *95%CI* [-0.68, 0.40], *df* = 64.43, *p* =.60).

The final analytic sample included *n* = 80 participants, of whom 53 identified as White, 2 as Black/African American, 4 as Hispanic/Latina, 1 as Asian, and 20 as Multiracial (see Table 1). The participants’ mean age at baseline was approximately 15.5 years old (*SD* = 1.16) and their average age at the emerging adult interview (T2) was approximately 24 years old (*SD* = 2.48). Most participants identified as heterosexual (*n* = 58). Participants’ partners were predominantly male and tended to be older, with a mean age of 28 years (*SD* = 7.04) at the emerging adult interview. The intimate partnerships tended to be long-term, with the average relationship duration at the emerging adult interview (T2) being over two years (*M* = 910.91 days, *SD* = 853.38).

**Table 1:** Participant and partner characteristics

Characteristic	Mean (Range) or %	*N*
Participant age at baseline (T1)	15.46 (12.95, 17.80)	80
Participant age at T2 interview	23.98 (19.11, 30.44)	80
Participant Sexual Orientation		79
Heterosexual Homosexual Bisexual Not sure	73.42% 1.27% 21.52% 3.80%	58 1 17 3
Partner age at T2 interview	28.46 (19, 61)	79
Partner Sex		79
Female	6.33%	5
Male	93.67%	74

Note. The total *N*s for participant sexual orientation, partner age, and partner sex do not equal 80 due to missing values on these variables

At baseline (T1) participants reported the frequency of their past 12-month alcohol use. During emerging adulthood (T2), participants were asked if they were currently involved with anyone and/or if they had a romantic relationship in the past six months, as well as the duration of their intimate partnership if one was reported. If they reported having a partner and consuming alcohol within the past six months, they then reported on their past six-month alcohol use and their intimate partner’s encouragement of their alcohol use on an 11-item subscale (Online Resource 1) during the T2 interview. At an assessment approximately six months after the T2 interview, participants reported their past six-month alcohol use frequency via a telephone interview (T3).

### Measures

#### Intimate Partner Encouragement of Alcohol Use (T2)

Intimate partner encouragement of alcohol use was collected using a subscale from a measure developed at the Oregon Social Learning Center (Oregon Social Learning Center, 2003). Participants were asked questions such as “How much does your partner approve of your drinking alcohol?”. Three items on the 11-item subscale were removed because they did not measure the partner’s encouragement of alcohol use directly. The remaining scale items lacked internal reliability ($\:\text{{\alpha}}\text{}\text{=}\text{}\text{0.55}$). To correct this, scale items were systematically deleted to reach a Cronbach’s alpha value of 0.70, the acceptable level for an exploratory scale (Nunnally, 1978). After three scale items were removed, five items remained to achieve a Cronbach’s alpha value of 0.71. In addition to the item noted above, the four remaining scale items asked participants to rate how often over the past six months (from “never” to “often”) their partner (1) picked up alcohol for them at the store, (2) offered them alcohol when they were together, (3) tried to be sure that there was always a supply of alcohol when they were together, and (4) encouraged them to drink until they got drunk. The mean of the five included scale items was used to create the T2 partner encouragement of alcohol use score, with higher scores indicating more encouragement of alcohol use.

#### Outcome: Alcohol Use (T3)

The primary outcome alcohol use variable was collected by asking participants how many times they drank beer, wine, or hard liquor in the past six months. These data were collected at T3, which occurred approximately six months after the T2 interview. The raw frequency of drinking occasions that participants reported was used in this analysis as the dependent variable.

#### Covariates

The participants’ age at the T2 interview was used to control for participant age differences, given variability of age at study enrollment and time between enrollment and the T2 assessment. Intervention condition was randomly assigned in the parent study and was coded 0 (control/Group Care treatment) and 1 (TFCO). It was included as a covariate to control for intervention effects on alcohol use because the intervention addressed risky substance use. To measure baseline alcohol use at study entry (T1), participants were asked how many times they drank beer, wine, or hard liquor in the past 12 months. Self-reported T1 alcohol use was coded as 1 = never, 2 = tried once or twice, 3 = occasionally (3–51 times), 4 = 1–6 times/week, 5 = 1 or more times/day in the past 12 months. Participants self-reported their past-six-month alcohol use at the emerging adult interview (T2), which was measured using the raw frequency of drinking occasions and added to the model to adjust for differences in alcohol use frequency at T2. Relationship duration was measured in days and added to the model as a covariate to account for the potentially varying effect of encouragement on alcohol use depending on the length of time the participant had been with their partner at T2. Because 12 participants’ emerging adult interview (T2) was their final study visit and other participants had varying amounts of time between T2 and T3 (range = 0-407 days), the number of days between T2 and T3 was added to the model to account for the time between participants’ report of their partner’s encouragement (T2) and their outcome alcohol use (T3). All covariates, with the exception of intervention condition, were z-scored.

### Analytic Approach

A negative binomial model was used to assess the relationship between intimate partner encouragement of alcohol use (T2) and alcohol use in emerging adulthood (T3), controlling for participants’ age at the T2 interview, intervention condition, T1 alcohol use, T2 alcohol use, relationship duration, and days between T2 and T3. A negative binomial model was selected because it is flexible enough to accommodate count data that are overdispersed and contain excess zeros, all of which were features of the alcohol use frequency outcome variable in this study. Because negative binomial models may be biased in small sample sizes, key model assumptions were rigorously evaluated using a Kolmogorov-Smirnov (KS) test, dispersion test, and outlier test, and results indicated a negative binomial model was statistically appropriate. To account for variability in alcohol use over time, participants’ alcohol use frequency in adolescence (T1) was included in the model as a covariate, along with their alcohol use at T2, age at T2, intervention condition in the parent study, partnership duration, and the days between T2 and T3.

The dataset was loaded into R 4.2.1 (R Core Team, 2024) and ineligible participants were removed. Sixty-six of the remaining cases contained no missing data and an additional 14 cases were missing data on one variable. Twelve of these cases were missing T3 alcohol use because their T2 interview was their final study visit. Because the time between T2 and T3 was adjusted for and these participants reported their alcohol use at T2, their T2 alcohol use was also used as their T3 alcohol use in the complete case analysis to maximize statistical power. In the imputed analysis, T3 alcohol use was treated as missing for the cases whose final study visit occurred at T2. All 14 cases with missing data were multiply imputed using the mice package in R (van Buuren & Groothuis-Oudshoorn, 2011). The classification and regression tree (CART) method was used to estimate 20 imputed datasets with a maximum of 10 iterations. Model estimates were pooled across the 20 imputed datasets using Rubin’s rules to obtain final results (Rubin, 1987). The MASS (Venables & Ripley, 2002) and lme4 (Bates et al., 2015) packages were used to fit a negative binomial model to the complete cases (*n* = 78) and then another negative binomial model was fit to the imputed dataset (*n* = 80). Results from the complete case and imputed data were compared.

## Results

T2 alcohol use was the only covariate in the model that was significantly correlated with T3 alcohol use (see Table 2). The imputed values fit the existing data well (Online Resource 2), as did the negative binomial model (Online Resource 3). The results of the negative binomials performed on the complete case analysis and the imputed dataset were similar in terms of magnitude, but not statistical significance. Age and T2 alcohol use were significantly associated with T3 alcohol use in the complete case analysis, but not the imputed analysis (see Table 3). This likely occurred because T2 alcohol use was carried over to T3 for participants whose final study visit occurred at T2 (*n* = 12) in the complete case analysis, resulting in a stronger association with the participant’s prior alcohol use for these cases than the multiple imputation estimated. To ensure that this did not bias results, a sensitivity analysis was conducted with only cases that had no missingness when T2 alcohol use was not carried over to T3 (*n* = 66; see Table 4). This sensitivity analysis estimated that for each standard deviation increase in partner encouragement of alcohol use, participants’ rate of T3 alcohol use frequency increased by 144% (*IRR* = 2.44, *95% CI* [1.45, 4.22], *p* <.001). In this model, T2 alcohol use and age were no longer significant predictors of T3 alcohol use, indicating that the complete case analysis may have been biased by the cross-sectional cases.

**Table 2:** Correlations of major study variables

Variable	M	SD	1	2	3	4	5	6	7	8
1. Age at T2 interview	23.98	2.48	-							
2. Intervention Condition	N/A	N/A	-0.02	-						
3. Relationship Duration	910.91	853.38	0.15	0.08	-					
4. Baseline (T1) Alcohol Use	3.09	1.31	-0.03	-0.03	0.10	-				
5. T2 Alcohol Use	17.59	35.97	0.10	0.19	-0.15	-0.01	-			
6. Partner Encouragement of Alcohol Use (T2)	1.88	0.62	0.09	-0.11	-0.09	0.00	0.30**	-		
7. Days between T2 and T3	161.35	83.73	0.32**	-0.05	0.09	-0.02	-0.03	-0.15	-	
8. Alcohol Use (T3)	17.01	39.55	0.09	0.06	-0.02	0.18	0.46***	0.31*	-0.18	-

Note. *p <.05, **p <.01, ***p <.001

Intervention Condition coded 0 = control/Group Care treatment, 1 = Treatment Foster Care Oregon treatment. Baseline alcohol use (T1) was coded as 1 = never, 2 = tried once or twice, 3 = “Occasionally”, 4 = 1–6 times/week, 5 = 1 or more times/day. Possible values for partner encouragement of alcohol use ranged from 1–4, with 4 being the highest level of encouragement for alcohol use

**Table 3:** Negative binomial regression results for the complete case analysis and the imputed dataset

Predictor variable	Complete case analysis (*n* = 78)		Imputed dataset (*n* = 80)	
	IRR	95%CI	IRR	95% CI
Intercept	5.00***	2.95, 9.00	8.26***	3.09, 22.13
Age	1.55*	0.98, 2.49	1.52	0.91, 2.54
Intervention Condition	1.99	0.91, 4.42	1.44	0.60, 3.46
Relationship Duration	1.09	0.72, 1.74	0.94	0.58, 1.52
T1 Alcohol Use	1.48*	1.03, 2.12	1.61*	1.03, 2.51
T2 Alcohol Use	1.48*	1.01, 2.40	1.42	0.91, 2.23
Days between T2 and T3	0.70	0.46, 1.05	0.53	0.14, 1.99
T2 Partner Encouragement of Alcohol Use	2.24***	1.47, 3.50	2.09*	1.14, 3.82
AIC	479.69		519.82	
BIC	500.91		541.25	

Note. *p <.05, **p <.01, ***p <.001

All predictor variables were z-scored except for intervention condition. Intervention Condition coded 0 = control/Group Care treatment, 1 = Treatment Foster Care Oregon treatment. Baseline alcohol use was coded as 1 = never, 2 = tried once or twice, 3 = “Occasionally,” 4 = 1–6 times/week, 5 = 1 or more times/day. T2 alcohol was used as the outcome variable for participants in the complete case analysis if T2 was their last study visit (*n* = 12) and thus, the correlation between T2 alcohol use and the outcome should be interpreted with caution. Possible values for partner encouragement of alcohol use ranged from 1–4, with 4 being the highest level of encouragement for alcohol use

**Table 4:** Negative binomial regression results for the complete case analysis excluding cross-sectional cases

Predictor Variable	IRR	95% CI
Intercept	4.75***	2.42, 10.18
Age	1.50	0.88, 2.60
Intervention Condition	2.32	0.92, 5.97
Relationship Duration	1.04	0.66, 1.77
T1 Alcohol Use	1.52*	0.99, 2.27
T2 Alcohol Use	1.35	0.90, 2.26
Days between T2 and T3	0.66	0.32, 1.53
T2 Partner Encouragement of Alcohol Use	2.44***	1.45, 4.22
AIC	396.87	
BIC	416.58	

Note. *p <.05, **p <.01, ***p <.001

All predictor variables were standardized except for intervention condition. Intervention Condition coded 0 = control/Group Care treatment, 1 = Treatment Foster Care Oregon treatment. Baseline alcohol use was coded as 1 = never, 2 = tried once or twice, 3 = “Occasionally”, 4 = 1–6 times/week, 5 = 1 or more times/day. Possible values for partner encouragement of alcohol use ranged from 1–4, with 4 being the highest level of encouragement for alcohol use

In the imputed analysis, T3 alcohol use was treated as missing for participants who did not have a study visit after T2 and multiple imputation was used to estimate T3 alcohol use for those cases. The negative binomial performed on the imputed dataset estimated that, on average, for each standard deviation increase in partner encouragement of alcohol use, participants’ rate of T3 alcohol use frequency increased by 109% (*IRR* = 2.09, *95% CI* [1.14, 3.82], *p* =.02). The imputed analysis accurately reflected the missingness on T3 alcohol use, used the largest number of cases (*n* = 80), and yielded the most conservative estimate of the effect of intimate partner encouragement on alcohol use.

## Discussion

The current study used data from a longitudinal, prospective study of females formerly involved with the U.S. JJ system to investigate the role of intimate partner encouragement of alcohol use on alcohol use from adolescence to emerging adulthood. Participants reported on their intimate partners’ encouragement of their alcohol use during emerging adulthood (T2), and then six months later, they reported on the frequency of their own alcohol use (T3). We found that having greater partner encouragement of alcohol use was significantly associated with greater alcohol use frequency approximately six months later. These findings have implications for prevention because they indicate that encouragement of alcohol use by a romantic partner may be a risk factor for alcohol use among females with a history of involvement in the JJ system.

Because the duration and seriousness of romantic relationships tends to increase in emerging adulthood (Arnett, 2000), intimate partners play an integral role in shaping substance use trajectories throughout and beyond this developmental period (Blumenstock & Papp, 2021). Additionally, alcohol use peaks during emerging adulthood (Center for Disease Control and Prevention, 2022a), making it a critical time to identify risk factors for alcohol consumption. Given this confluence of factors, it is unsurprising that intimate partner encouragement of alcohol use is associated with a greater frequency of alcohol use among this population.

Interventions to reduce alcohol use among emerging adult females exposed to high levels of adversity are needed to mitigate the risk of recidivism (Andrews et al., 2012) and the myriad of health effects associated with heavy drinking, which disproportionately affect females (Center for Disease Control and Prevention, 2022b). Although these findings do not establish that intimate partner encouragement of alcohol use can be changed, it minimally establishes the need to measure intimate partner encouragement of alcohol use when conducting alcohol-related research in this population. Further research is needed to examine whether intimate partners can learn how to effectively discourage alcohol use and what forms of discouragement are effective. If it is possible to intervene on the amount of encouragement of alcohol use provided by an intimate partner, this may be an effective supplement to existing preventive interventions to reduce alcohol use in female emerging adults.

This study fills a gap in the existing literature by examining how intimate partner encouragement of alcohol use across multiple domains associates with alcohol use in emerging adulthood. Our findings shed light on a potential risk factor for a population that suffers many alcohol-related consequences. These findings contradict previous research that did not find a significant effect of intimate partner encouragement of alcohol use on alcohol use among emerging adult drinkers (Cheong et al., 2021), likely because we utilized a longitudinal design, assessed several dimensions of partner encouragement of alcohol use, and limited our sample to females who were formerly involved in the U.S. JJ system. Our foundational study highlights a potential need for the development of interventions that include and/or focus on partners of emerging adults who may be at higher risk for problematic alcohol use. It also emphasizes the need to develop accurate measures of partner encouragement of alcohol use and a better understanding of the complex ways in which intimate partners can influence alcohol use behaviors. Furthermore, these analyses should be expanded beyond our sample of predominantly White, heterosexual females who were formerly involved with the U.S. JJ system.

### Limitations

Our longitudinal design came with the downside of having a lengthy recall period for alcohol use that varied between the baseline (T1) alcohol use measure and the emerging adult interview (T2) and outcome (T3) alcohol use measure (12 months and six months, respectively). Although the baseline measure captured seasonal variation in drinking, a 12-month recall period is likely too long to result in a precise measure of alcohol use. The T2 and T3 alcohol use measures also required participants to think back over an extended period of time, though the recall period used for T2 and T3 was half as long (six months). Self-reported alcohol use is subject to desirability bias, which would have been mitigated if a biomarker of alcohol use was also collected. T1 alcohol use was coded as an ordinal variable rather than as a continuous measure because the raw frequency data were not available for all participants at baseline. To ensure this ordinal coding did not bias the results, dummy-coded variables representing each level of baseline alcohol use were used in a separate sensitivity analysis. An ANOVA indicated that this alternative model fits the data significantly better than the model with baseline alcohol use measured ordinally ($\:{\chi\:}^{2}$(1) = 4.32, *p* =.04). However, the ordinal model had a lower AIC (dummy-coded model: *AIC* = 482, ordinal-coded model: *AIC* = 479.7), indicating that the increased complexity of the dummy-coded model outweighs the improvements to model fit. A similar pattern of results emerged substantively and in terms of statistical significance in both models, except for intervention condition, which became significant when baseline alcohol use was dummy-coded, and age, which was no longer significant in the dummy-coded model. For parsimony, the ordinal measure of baseline alcohol use was retained. Furthermore, this measure of alcohol use captured the frequency of drinking occasions but not the quantity of alcohol consumed during that period.

At T3, 20 participants reported that they were no longer with the same partner who they reported on at T2. While most couples included in this study remained together between T2 and T3 (*n* = 60), those that did not likely had less exposure to their partner’s encouragement of their alcohol use which may have affected their alcohol use at T3. Participants who ended their relationship between T2 and T3 could have entered a new relationship with a partner who encouraged their alcohol use differently than the partner they reported on at T2.

Additionally, participants were asked to report on specific encouraging behaviors, which may not have adequately captured all of the partners’ encouraging behaviors and/or the intent behind their actions. The measure used to capture intimate partner encouragement of alcohol use needed to be adjusted due to low internal reliability, which may point to inconsistencies in the types of encouragement of alcohol use provided by partners. Although we did find a correlation between intimate partner encouragement of alcohol use and alcohol use, this analysis is not sufficient evidence of a causal relationship between these two variables. It is likely that additional factors influenced this finding such as the role of partner selection, use of alcohol to cope with trauma, and encouragement of alcohol use from other significant people in their social network.

### Future Directions

Future research should focus on developing a standardized measure of alcohol encouragement that is validated across a range of populations and ages. Evaluating which types of encouraging behaviors are most influential on alcohol use and how partners of high-risk drinkers can be taught to avoid such behaviors to help their partner decrease their alcohol use is another important future direction. Because the participants in the current study were all female and the majority had male partners (94%), further research is needed to demonstrate the effect of partner encouragement of alcohol use on alcohol use in males and in females whose partners are not male. Given that prior research found that participants who are highly satisfied in their relationships may be more receptive to alcohol-related cues from their partners (Fleming et al., 2010), future research should investigate the impact of relationship quality on the effect of intimate partner encouragement of alcohol use and account for the role that substance use plays in partner selection.

## Data Availability

The data and code used in the current research cannot be publicly shared but are available upon request. The data and/or code can be obtained by emailing: aturner8@uoregon.edu.
