# Correction: The Association Between Intimate Partner Encouragement of Alcohol Use and Alcohol Use Among Females Formerly Involved in the Juvenile Justice System

**DOI:** 10.1007/s10935-025-00837-y

**Published:** 2025-04-11

**Authors:** Avery Turner, Diana Jenkins, Maria Schweer-Collins, Leslie D. Leve

**Affiliations:** 1 College of Education, University of Oregon, Eugene, USA; 2 Prevention Science Institute, University of Oregon, Eugene, USA; 3 Department of Psychology, Iowa State University, Ames, USA

**Correction to: Journal of Prevention** 10.1007/s10935-025-00828-z

The article “The Association Between Intimate Partner Encouragement of Alcohol Use and Alcohol Use Among Females Formerly Involved in the Juvenile Justice System”, written by Avery Turner, Diana Jenkins, Maria Schweer-Collins, and Leslie D. Leve was originally published under exclusive license to Springer Science+Business Media, LLC, part of Springer Nature. As a result of the subsequent decision to publish the article under the open access model, the article’s copyright notice was changed on 12 March 2025 to © The Author(s) 2025 and the article is now distributed under a Creative Commons Attribution 4.0 International License, which permits use, sharing, adaptation, distribution and reproduction in any medium or format, as long as you give appropriate credit to the original author(s) and the source, provide a link to the Creative Commons licence, and indicate if changes were made. The images or other third party material in this article are included in the article’s Creative Commons licence, unless indicated otherwise in a credit line to the material. If material is not included in the article’s Creative Commons licence and your intended use is not permitted by statutory regulation or exceeds the permitted use, you will need to obtain permis?sion directly from the copyright holder. To view a copy of this licence, visit http://creativecommons.org/licenses/by/4.0/.

